# Constructing High-Transparency, Self-Healing and Reprocessable Poly(thiourethane) Elastomers Based on Zn^2+^-Multidentate Pyrimidine Coordination

**DOI:** 10.3390/polym18151910

**Published:** 2026-08-04

**Authors:** Na Wei, Hanxu Zhu, Bing Li, Weijun Yang

**Affiliations:** 1 The Key Laboratory of Synthetic and Biological Colloids, Ministry of Education, School of Chemical and Material Engineering, Jiangnan University, Wuxi 214122, China; 6240608018@stu.jiangnan.edu.cn (N.W.); zhuhanxu@nimte.ac.cn (H.Z.); libing_73@163.com (B.L.); 2 Key Laboratory of Bio-Based Polymeric Materials Technology and Application of Zhejiang Province, Ningbo Institute of Materials Technology and Engineering, Chinese Academy of Sciences, Ningbo 315201, China; 3 Collaborative Innovation Center of Chemical Science and Engineering, Key Laboratory for Green Chemical Technology of Ministry of Education, School of Chemical Engineering and Technology, Tianjin University, Tianjin 300072, China; 4 Zhejiang Rongsheng Environmental Protection Paper Co., Ltd., No. 588 East Zhennan Road, Pinghu Economic Development Zone, Jiaxing 314213, China

**Keywords:** polythiourethane elastomers, metal coordination, self-healing, transparency, reprocessability

## Abstract

To develop self-healing polyurethane materials with high transparency and superior mechanical performance, in this work, the poly(thiourethane) elastomers were prepared by incorporating the dynamic thiourethane bonds via thiol–isocyanate click reaction, followed by the addition of 1-(3-aminopropyl)imidazole (IZ), 3-hydroxypyridine (HP), and 2,4-diamino-6-hydroxypyrimidine (HPM) as ligands to produce three different polyurethane networks (named PTU-IZ, PTU-HP, and PTU-HPM). Zinc chloride (ZnCl_2_) was further introduced to construct metal-coordinated crosslinking networks, recorded as PTU-IZ-Zn, PTU-HP-Zn, and PTU-HPM-Zn, respectively. The effects of ligands and Zn^2+^ coordination on the materials’ optical transmittance, mechanical properties, self-healing capability, and reprocessability were systematically investigated. The results demonstrate that HPM and Zn^2+^ will facilitate the formation of more effective crosslinking, which significantly enhances the mechanical properties of PTU-HPM from 4.61 MPa up to 9.04 MPa (PTU-HPM-Zn), while maintaining high transparency (89.0% light transmittance at 650 nm). Self-healing tests reveal that the PTU-HPM-Zn scratches can fully repair within 4 h at 70 °C. Reprocessability tests demonstrate that the internal crosslinked network of the material undergoes reversible dissociation, enabling a topological transition from a crosslinked to a linear structure and thereby imparting excellent thermal reprocessability. This study provides novel insights for the design and fabrication of high-performance transparent self-healing polyurethane materials.

## 1. Introduction

Polyurethane (PU) demonstrates promising potential for applications spanning flexible electronics, biomedicine, optical coatings, and other emerging fields, owing to its exceptional structural tailorability, tunable mechanical properties, and versatile processability [1,2,3,4]. Among these, transparent polyurethane elastomers—combining high optical clarity with inherent flexibility—have emerged as pivotal materials for advanced applications such as flexible displays, optical lenses, and smart windows [5,6,7,8]. However, during long-term service, polyurethane elastomers are prone to microcracking induced by mechanical impact, chemical corrosion, and thermal aging. Such microcracks can severely compromise the optical transparency, degrade the mechanical integrity, shorten the service life, and ultimately limit reliability in demanding optical applications [9,10,11,12]. Imparting intrinsic self-healing ability to materials is a promising approach to improving the durability and extending the service life [13,14,15,16]. Therefore, the development of novel polyurethane elastomers that synergistically integrate high transparency, robust mechanical strength, and efficient self-healing has emerged as a central research focus.

The incorporation of dynamic crosslinks represents the primary strategy for imparting intrinsic self-healing properties to polyurethanes. This is predominantly achieved through the introduction of either dynamic covalent bonds—such as disulfides [17,18], imines [19,20], and Diels–Alder adducts [21,22]—or dynamic non-covalent interactions, including hydrogen bonds [23,24] and metal–ligand coordination [25,26,27]. Among these, self-healing polyurethanes based on dynamic covalent bonds typically rely on reversible chemical reactions between functional groups located on the polymer backbone or side chains, with the healing process driven by external stimuli such as light, heat, electricity, magnetic fields, or pH [28,29,30]. For instance, Fang et al. [31] synthesized a novel diol containing Diels–Alder (DA) bonds from *N*-(2-hydroxyethyl)maleimide and furfuryl alcohol, which was subsequently used to fabricate a waterborne polyurethane (WPU-DA-x). This material achieved complete crack healing within 30 min at 130 °C, exhibiting a repair efficiency as high as 92.5%. In another study, Xu et al. [32] prepared a prepolymer by reacting 2,2′-hydroxyethyl disulfide (HEDS) with isophorone diisocyanate (IPDI), followed by crosslinking with triethanolamine to obtain a photo-responsive polyurethane. Benefiting from the high mobility of aliphatic segments and the electron-donating effect of amide groups, this material combines high light transmittance (facilitating light penetration) and excellent mechanical properties (Young’s modulus of 5.04 MPa, tensile strength of 9.67 MPa) and achieves a high healing efficiency of 96% at 45 °C under natural light irradiation.

In contrast to dynamic covalent bonds, dynamic non-covalent bonds feature lower bond energy, enabling elastomers constructed via such interactions to achieve spontaneous healing more readily at room temperature. For example, inspired by the molecular structure of titin and the zinc-ion crosslinking mechanism in Nereis jaws, Xu et al. [33] designed a supramolecular polyurethane (SPU) elastomer integrating hierarchical hydrogen bonds and Zn^2+^-ligand coordination bonds. Within this system, hierarchical hydrogen bonds—ranging from single to quadruple, formed by urethane, urea, and UPy moieties—act synergistically with Zn^2+^ coordination to endow the material with excellent comprehensive properties. Leveraging a bilayer structure with a “soft core and hard shell” design, the elastomer reached a self-healing efficiency of 95% after 24 h of healing at room temperature.

However, a trade-off between mechanical properties and self-healing efficiency has generally existed in current systems: highly reversible dynamic crosslinking networks often exhibit insufficient mechanical properties, which are not conducive to achieving a high performance of elastomer materials. As a new type of dynamic covalent bond, thiourethane bonds (-S-C(=O)-NH-) possess low bond energy (approximately 218 kJ·mol^−1^) and can undergo reversible exchange under mild conditions (50–80 °C). Moreover, they can be efficiently incorporated into the polymer backbone via thiol–isocyanate click chemistry, which exhibits advantages such as high reaction efficiency, good selectivity, and few side reactions [34]. Thus, thiourethane bonds provide an ideal platform for constructing intrinsic self-healing systems. The synergistic integration of dynamic thiourethane bonds and metal coordination bonds is expected to realize multiple responses and the performance complementarity of dynamic networks at the molecular scale, thereby synergistically optimizing the mechanical strength, self-healing efficiency, and optical properties of materials. Meanwhile, metal–ligand coordination bonds provide a new approach for constructing crosslinked networks due to their advantages such as a tunable bonding strength, dynamic reversibility, and flexible structural design [35,36]. Among various metal ions, Zn^2+^ has attracted considerable attention due to its low toxicity, coordination reversibility, and good coordination ability with N/O-containing donors [37]. However, traditional monodentate ligands (e.g., imidazole, pyridine) often suffer from limited coordination capability, interference from counterions, and insufficient network stability. Moreover, phase separation is easily induced in composite materials, which impairs transparency [38,39,40]. Therefore, the design of new multidentate ligands to construct stable, uniform, and dynamically reversible coordination networks is the key to developing high-performance transparent self-healing polyurethanes.

Based on the aforementioned research background and existing challenges, this study proposes a synergistic design strategy integrating “dynamic thiourethane backbones with Zn^2+^-multidentate pyrimidine coordination”. Initially, a polythiourethane prepolymer was synthesized via thiol–isocyanate click reaction using poly(tetramethylene ether)diol (PTMG-1000), isophorone diisocyanate (IPDI), and 2,2′-(ethylenedioxy)diethanethiol (TGLDM) as raw materials. Subsequently, three different ligands—1-(3-aminopropyl)imidazole (IZ), 3-hydroxypyridine (HP), and 2,4-diamino-6-hydroxypyrimidine (HPM)—were introduced to prepare PTU-IZ, PTU-HP, and PTU-HPM polymer series, respectively. Metal-coordinated crosslinked networks were then constructed using zinc chloride (ZnCl_2_), yielding three composite materials: PTU-IZ-Zn, PTU-HP-Zn, and PTU-HPM-Zn. The effects of the ligand structure (monofunctional IZ and HP vs. multifunctional HPM) and their coordination with Zn^2+^ on the microstructure, optical properties, and mechanical behavior of the polythiourethanes were systematically investigated, with particular emphasis on elucidating the mechanism by which the multifunctional ligand–Zn^2+^ coordination in PTU-HPM-Zn contributes to its self-healing efficiency and reprocessability.

## 2. Materials and Methods

### 2.1. Materials

Zinc chloride (ZnCl_2_, 98%), 1-(3-aminopropyl)imidazole (IZ, ≥97%), 2,4-diamino-6-hydroxypyrimidine (HPM, ≥98%), isophorone diisocyanate (IPDI, 99%), N,N-dimethylacetamide (DMAc, 99%), and dibutyltin dilaurate (DBTDL, 95%) were purchased from Shanghai Maclin Biochemical Technology Co., Ltd. (Shanghai, China). 3-Hydroxypyridine (HP, 98%), poly(tetramethylene ether) diol (PTMG-1000, Mn = 1000 g·mol^−1^), 2,2′-(diethoxy)diethanethiol (TGLDM, ≥97%), and N,N-diisopropylethylamine (DIPEA, 99%) were obtained from Shanghai Aladdin Biochemical Technology Co., Ltd. (Shanghai, China). All reagents were used without further purification.

### 2.2. Preparation of Polyurethane Films and Zn^2+^-Coordinated Composites

Synthesis of PTU-IZ, PTU-HP, and PTU-HPM Films: First, 4.00 g of PTMG was weighed into a three-necked flask, dried under vacuum at 100 °C for 1 h, and then cooled to 70 °C. Then, 1.79 g of IPDI and two drops of DBTDL catalyst were added, followed by dissolving in 3 g of DMAc. The reaction was carried out at 70 °C for 2.5 h under nitrogen protection to obtain the prepolymer. Subsequently, 0.364 g of TGLDM and two drops of DIPEA catalyst were added and dissolved in 6 g of DMAc, and the chain extension reaction was performed at 65 °C for 8 h to obtain the polythiourethane (PTU) solution. Then, 0.266 g of IZ, 0.380 g of HP, and 0.168 g of HPM were added separately, and the reaction was continued at 60 °C for 12 h. The resulting solution was poured into a mold, volatilized at room temperature for 24 h, and then dried at 50 °C to obtain PTU-IZ film, PTU-HP film, and PTU-HPM film.

Preparation of Zn^2+^-Coordinated Composites (PTU-IZ-Zn, PTU-HP-Zn, PTU-HPM-Zn): The above three polyurethane films were weighed separately and dissolved in 10 g of DMAc, followed by stirring at 60 °C under nitrogen protection until completely dissolved. According to the molar ratio of Zn^2+^ to ligand of 1:1, the corresponding mass of ZnCl_2_ was weighed, dissolved in 2 g of DMAc, added to the above solution, ultrasonically dispersed for 5 min, and then stirred at 50 °C for 12 h. The solution was poured into a mold, volatilized at room temperature for 24 h, and dried at 50 °C for 24 h to obtain PTU-IZ-Zn, PTU-HP-Zn, and PTU-HPM-Zn composites, respectively.

### 2.3. Characterization of Polyurethane Films and Zn^2+^-Coordinated Composites

Fourier transform infrared spectroscopy (FT-IR), proton nuclear magnetic resonance spectroscopy (1H NMR), and gel permeation chromatography (GPC) were used to characterize the chemical structure and molecular weight of the polymers. The light transmittance of the films was tested by an ultraviolet–visible spectrophotometer (UV-Vis). The tensile properties were measured using a universal testing machine. The surface scratch healing process was observed by a super depth-of-field three-dimensional microscope. Detailed experimental conditions and instrument parameters are provided in the Supporting Information.

## 3. Results and Discussion

### 3.1. Structural Characterization of PTU-IZ, PTU-HP, and PTU-HPM

**Figure 1:**
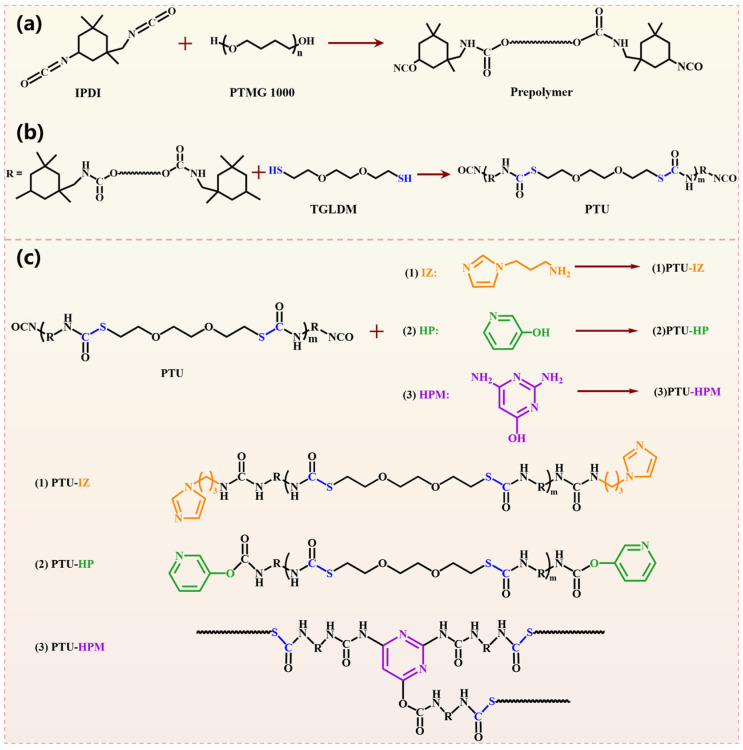
(**a**) Synthesis of the prepolymer; (**b**) introduction of thiourethane bonds; (**c**) synthesis of PTU-IZ, PTU-HP, and PTU-HPM.

The synthetic route of poly(thiourethane) elastomers is illustrated in Figure 1. Firstly, PTMG reacts with IPDI to form a prepolymer (Figure 1a); chain extension with TGLDM introduces dynamic thiourethane bonds (Figure 1b); finally, end-capping reactions are carried out with IZ, HP, and HPM, respectively, yielding PTU-IZ, PTU-HP, and PTU-HPM (Figure 1c).

**Figure 2:**
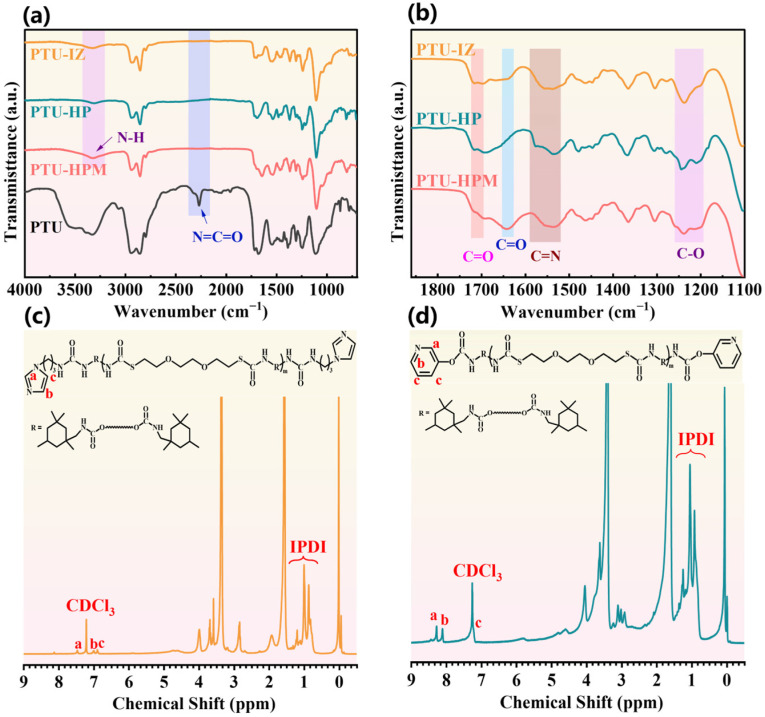
FT-IR spectra of PTU, PTU-IZ, PTU-HP, and PTU-HPM in the ranges of (**a**) 4000–700 cm^−1^ and (**b**) 1860–1100 cm^−1^; 1H NMR spectrum of (**c**) PTU-IZ; (**d**) PTU-HP. The labeled signals (a, b, and c) correspond to the protons marked in the molecular structures.

Figure 2a presents the FT-IR spectra of PTU-IZ, PTU-HP, PTU-HPM, and PTU in the wavenumber range of 4000–700 cm^−1^. Among them, the broad peak around 3300 cm^−1^ is attributed to the N-H stretching vibration absorption peak in the polyurethane molecular chain; the C-H stretching vibration peaks of methyl and methylene groups on IPDI are located at 2938 cm^−1^ and 2850 cm^−1^, respectively. In Figure 2b, the absorption peak at 1700 cm^−1^ is assigned to the stretching vibration of carbonyl (C=O) in urethane groups. At 1666 cm^−1^, characteristic absorption peaks of urea C=O are observed in both PTU-IZ and PTU-HPM, while no such peak is detected in PTU-HP, which is consistent with the structural differences of different end-capping ligands. The absorption peaks at 1555 cm^−1^ (PTU-IZ), 1573 cm^−1^ (PTU-HP), and 1557 cm^−1^ (PTU-HPM) correspond to the C=N vibration absorptions in the aromatic rings of imidazole, pyridine, and pyrimidine, respectively. The absorption band in the range of 1240~1200 cm^−1^ is the asymmetric stretching vibration peak of C-O-C bonds. During the end-capping reaction of PTU-HP and PTU-HPM, derived urethane groups with structures different from the direct reaction product of PTMG and IPDI are generated. The two urethanes in different environments correspond to different C-O-C bond vibration frequencies; thus, two sets of characteristic C-O-C absorption peaks appear in this range. The region below 1000 cm^−1^ is mainly the C-H out-of-plane bending vibration region [41]. In the infrared spectra of the three polyurethanes, no S-H absorption peak is observed at 2600 cm^−1^, indicating that TGLDM used as a chain extender has completely reacted. Meanwhile, no obvious N=C=O stretching absorption peak is detected at 2264 cm^−1^, suggesting that the free isocyanate groups in the system have been completely reacted. All the above analyses confirm the successful synthesis of the three polyurethanes.

1H NMR spectra further confirm the successful introduction of end-capping structures. PTU-IZ exhibits characteristic signals of imidazole ring protons in the range of δ = 6.92–7.48 ppm (Figure 2c), while PTU-HP shows characteristic signals of pyridine ring protons at δ = 7.08–8.24 ppm (Figure 2d). Due to the poor solubility of PTU-HPM in common deuterated solvents, no valid ^1^H NMR spectrum was obtained. GPC test results (Table S1, Figure S1) indicate that the number-average molecular weight (M_n_ = 47,730 g·mol^−1^) of PTU-HPM is significantly higher than that of PTU-IZ (33,500 g·mol^−1^) and PTU-HP (38,960 g·mol^−1^), which is consistent with the structural feature that HPM, as a multifunctional crosslinking agent, forms a lightly crosslinked network during the polymerization process.

### 3.2. Structural and Performance Characterization of PTU-IZ-Zn, PTU-HP-Zn, and PTU-HPM-Zn

**Figure 3:**
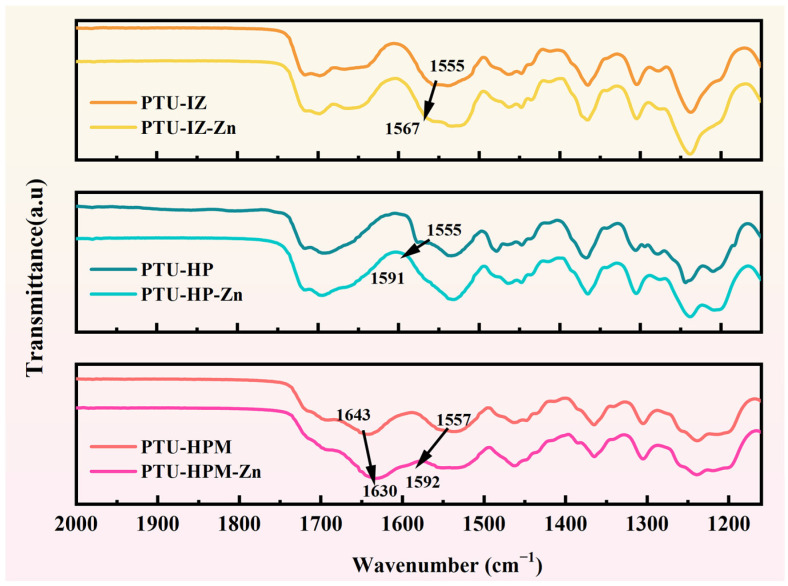
FT-IR spectra of PTU-IZ, PTU-IZ-Zn, PTU-HP, PTU-HP-Zn, PTU-HPM, and PTU-HPM-Zn in the wavenumber range of 2000–1160 cm^−1^.

To verify the formation of coordination interactions between Zn^2+^ and the ligands, the chemical structures of PTU-IZ-Zn, PTU-HP-Zn, and PTU-HPM-Zn were characterized using FT-IR spectroscopy, as shown in Figure 3. Upon coordination with ZnCl_2_, the characteristic C=N stretching vibration peaks of the ligand moieties in the polyurethane backbone exhibited a blue shift. Specifically, the absorption peaks corresponding to the C=N bonds in the imidazole, pyridine, and pyrimidine rings shifted from 1555 cm^−1^, 1573 cm^−1^, and 1557 cm^−1^ to 1567 cm^−1^, 1591 cm^−1^, and 1592 cm^−1^, respectively. This blue shift is consistent with the formation of coordination between Zn^2+^ and the ligand groups in all three composite systems, leading to the formation of stable coordination structures [37]. It is worth noting that only slight wavenumber shifts of the C=N characteristic absorption peaks are observed for PTU-IZ and PTU-HP, suggesting that no significant coordination interactions are formed between Zn^2+^ and ligands in these two systems. In addition, the characteristic absorption peak of urea C=O in PTU-HPM shifts red from 1643 cm^−1^ to 1630 cm^−1^. This phenomenon further supports that the oxygen atoms on the urea carbonyl groups in the PTU-HPM structure can also act as coordination sites to coordinate with Zn^2+^, forming complex multidentate coordination structures [42].

### 3.3. Mechanical Properties

**Figure 4:**
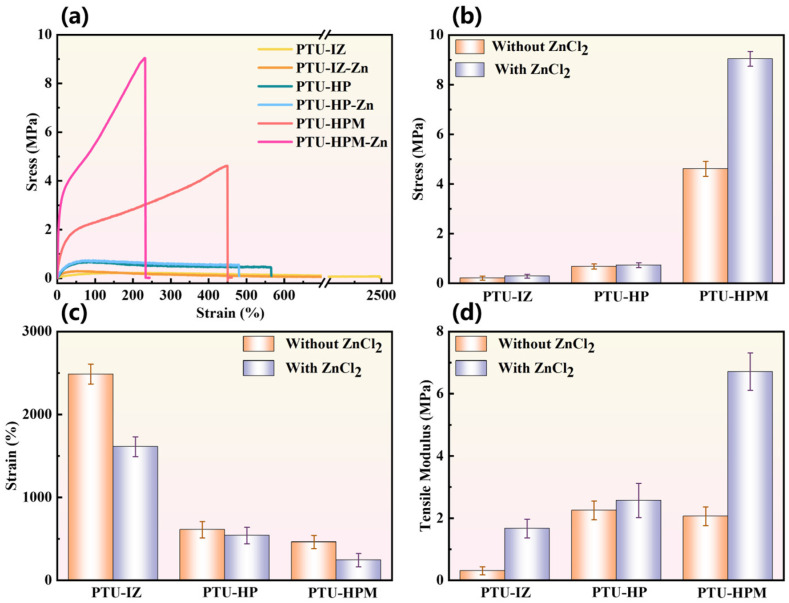
Tensile tests of polyurethane specimens: (**a**) stress–strain curves; (**b**) tensile strength at break; (**c**) elongation at break; (**d**) elastic modulus.

The mechanical properties of the polyurethanes PTU-IZ, PTU-HP, PTU-HPM, and their metal-coordinated counterparts PTU-IZ-Zn, PTU-HP-Zn, and PTU-HPM-Zn are shown in Figure 4. Linear polyurethanes PTU-IZ and PTU-HP both exhibit typical weak physical crosslinking characteristics, with tensile strengths of 0.21 MPa and 0.68 MPa and elongation at break as high as 2487% and 611%, respectively. This result stems from the lack of effective chemical crosslinking between the molecular chains of the two materials; during stretching, the structure is maintained only by intermolecular forces, leading to easy slippage of molecular chains and significantly low mechanical strength [43]. Upon introduction of Zn^2+^, only a marginal improvement in mechanical strength was observed for PTU-IZ-Zn and PTU-HP-Zn. This limited enhancement may be attributed to two factors. First, chloride ions remain coordinated to Zn(II) in solution, and coordination between Zn^2+^ and the terminal imidazole or pyridine ligands requires ligand exchange [44]. This process may reduce the effective coordination efficiency of Zn^2+^ with the polymer ligands. Second, compared with HPM, both imidazole and pyridine terminal groups pro-vide fewer coordination sites and therefore are less favorable for constructing an extended dynamic coordination network. As a result, PTU-IZ-Zn and PTU-HP-Zn mainly exhibit relatively weak coordination crosslinking and only limited improvements in mechanical properties. Notably, PTU-IZ-Zn exhibited a significantly higher elongation at break (2487%) compared to PTU-HP-Zn (611%). This difference is primarily due to the lower binding constant of the imidazole–Zn^2+^ coordination bond, which can dynamically break and reform under stress, thereby dissipating energy and enhancing material ductility [45].In contrast, the mechanical properties of PTU-HPM prepared by introducing the multifunctional ligand HPM are significantly enhanced, with a tensile strength of 4.61 MPa and an elongation at break of 462%. This is because HPM, as a multifunctional ligand, constructs a lightly chemically crosslinked network during the polymerization process, which effectively inhibits molecular chain slippage and improves the load-bearing capacity of the material [41]. After further introducing Zn^2+^, the tensile strength of PTU-HPM-Zn is highly increased to 9.04 MPa, while the elongation at break decreases to 244%. It is speculated that Zn^2+^ not only coordinates with the nitrogen atoms on the pyrimidine ring but also may form additional coordination bonds with the oxygen atoms in adjacent urea groups, constructing a denser and more stable metal–ligand crosslinked network [46]; this network improves the tensile strength of the material while restricting the free movement of molecular chain segments, leading to a decrease in ductility.

### 3.4. Optical Properties

**Figure 5:**
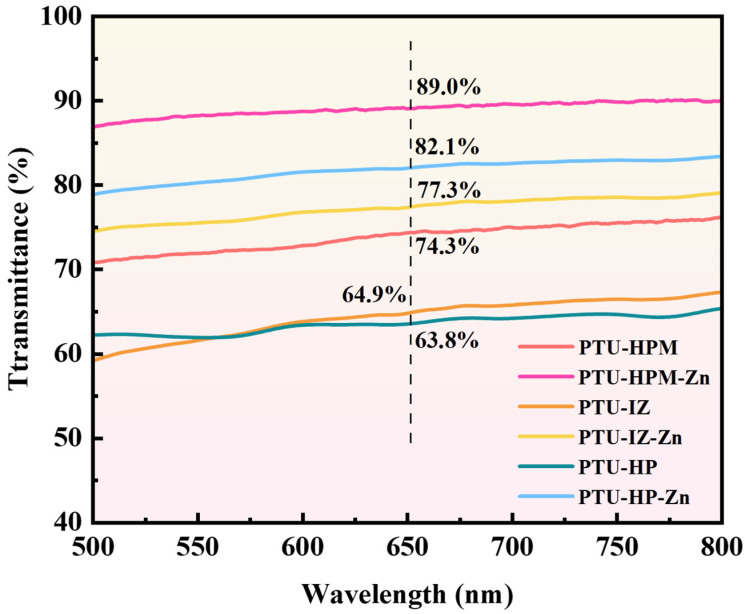
UV–Vis transmission spectra of PTU-IZ, PTU-IZ-Zn, PTU-HP, PTU-HP-Zn, PTU-HPM, and PTU-HPM-Zn.

To systematically evaluate the optical transparency of the materials, ultraviolet–visible (UV-Vis) transmission spectroscopy was employed to characterize the polyurethane samples. Figure 5 presents the resulting transmission spectra. PTU-IZ and PTU-HP, which are end-capped with IZ and HP ligands, exhibit similar light transmittance. In contrast, PTU-HPM, which incorporates the HPM crosslinker, displays significantly higher optical clarity, with a transmittance of 73.4% at 650 nm. This difference can be attributed to the lightly covalent crosslinked network formed in PTU-HPM: the crosslinked structure suppresses the hydrogen-bond-driven aggregation of hard segments and restricts their tight packing during microphase separation, thereby reducing the size of the dispersed domains and rendering a more uniform morphology [47]. Upon the introduction of ZnCl_2_, the light transmittance of all three metal-coordinated polyurethanes (PTU-IZ-Zn, PTU-HP-Zn, and PTU-HPM-Zn) improves significantly. Notably, PTU-HPM-Zn achieves a high transmittance of 89.0% at 650 nm. This enhancement is attributed to the formation of new coordination structures between Zn^2+^ and the ligand molecules, which further regulate microphase separation behavior and promote a more homogeneous distribution of smaller dispersed domains throughout the system [48]. These observations indicate that the dynamic coordination bonds formed between Zn^2+^ and the ligands not only reconstruct the original supramolecular interaction network but also further refine microphase separation—leading to finer domain sizes and improved spatial uniformity, thereby effectively reducing light scattering and enhancing the overall optical transparency [49].

### 3.5. Self-Healing Properties

**Figure 6:**
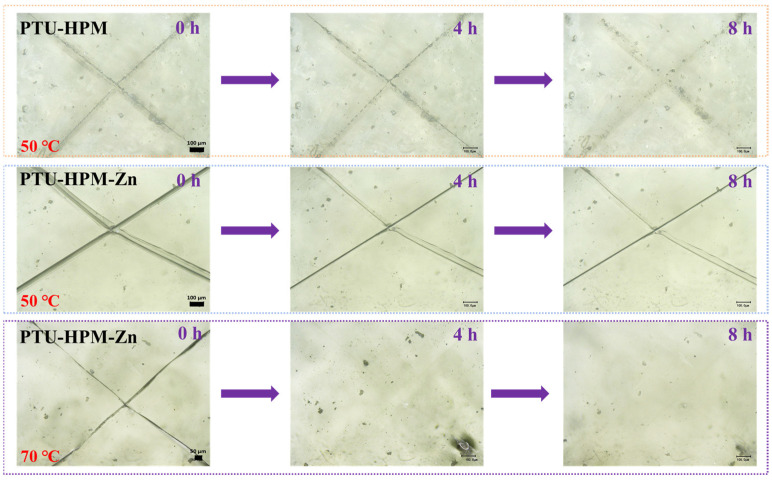
Surface scratch test of PTU-HPM at 50 °C and PTU-HPM-Zn at 50 °C and 70 °C.

Given the comprehensive advantages of the PTU-HPM system in both mechanical and optical properties, its self-healing and reprocessing capabilities were subsequently investigated. To gain insight into the self-healing behavior, surface scratches on PTU-HPM and PTU-HPM-Zn were observed using a super-depth-of-field 3D microscope at 50 °C and 70 °C, with the results presented in Figure 6. At 50 °C, the surface scratches on PTU-HPM exhibited a clear progressive healing trend: within 4 h, continuous fine scratches transformed into discontinuous fine lines; after 8 h, these further evolved into faint blurred traces, indicating substantial scratch recovery. This behavior demonstrates the material’s favorable chain mobility and efficient dynamic bond reorganization. In contrast, under the same temperature, the healing process of PTU-HPM-Zn was notably limited: although the scratch width narrowed significantly within 4 h, and shallow scratches were completely repaired, no obvious further morphological change occurred afterward, leaving deeper scratches incompletely healed. This phenomenon is attributed to the Zn^2+^-induced coordination crosslinking network. While enhancing the mechanical stability, the network restricts the freedom of polymer chain movement to some extent, hindering sufficient dissociation and reformation of reversible bonds, thereby slowing the self-healing kinetics [50].

Notably, when the healing temperature was raised to 70 °C, PTU-HPM-Zn achieved nearly complete scratch repair within 4 h. The relatively low bond energy of the thiourethane bonds, combined with the increased temperature, not only significantly accelerates the dissociation–reassociation kinetics of these bonds but also induces partial thermal dissociation of the metal–ligand coordination bonds. This balances sufficient crosslink density with restored segmental mobility, enabling efficient reconstruction of the dynamic network [51]. To quantitatively evaluate this self-healing capability, tensile recovery tests were conducted under the same healing conditions (70 °C, 4 h). The healing efficiency, calculated as η = σ_h_/σ_0_ × 100%, reached 87.1% based on the recovery of tensile strength from 9.04 MPa to 7.87 MPa (Figure S2, Supporting Information). This quantitative result further confirms that the synergistic effect of dynamic thiourethane bonds and reversible Zn^2+^–pyrimidine coordination effectively reconstructs the polymer network after damage. These results highlight the critical role of temperature in regulating the self-healing behavior of metal-coordinated polythiourethanes and provide a theoretical basis for achieving on-demand healing through thermal stimulation in practical applications.

### 3.6. Reprocessability

**Figure 7:**
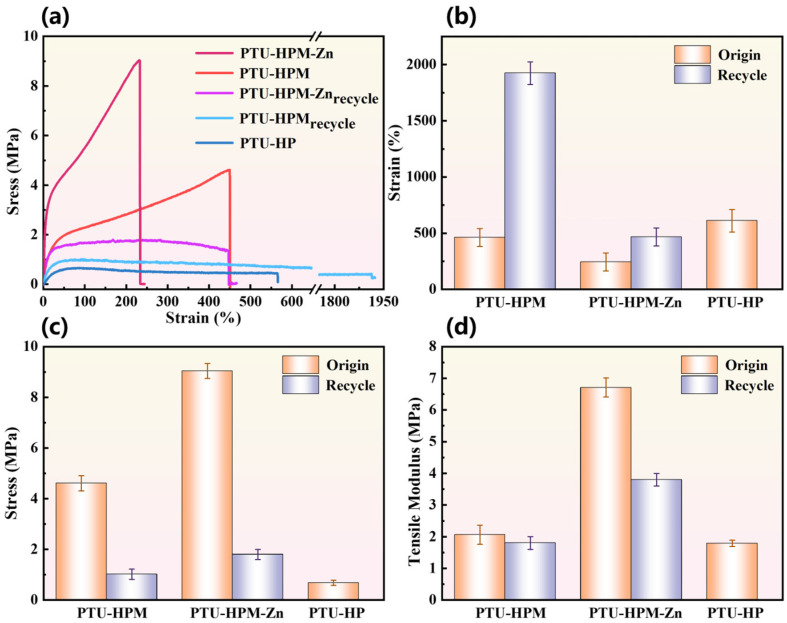
Tensile tests of polyurethane specimens after hot-pressing reprocessing: (**a**) stress–strain curves; (**b**) tensile strength at break; (**c**) elongation at break; (**d**) Young’s modulus.

In this study, mechanical property tests were re-conducted on the hot-pressed reprocessed samples of PTU-HPM and PTU-HPM-Zn, with the results shown in Figure 7. Under hot-pressing conditions (120 °C, 10 MPa), the mechanical properties of the samples changed significantly: the tensile strengths of PTU-HPM and PTU-HPM-Zn dropped to 1.02 MPa and 1.80 MPa, respectively, from their original values of 4.61 MPa and 9.04 MPa. In contrast, the elongation at break showed substantial improvement, increasing from 462% to 1924% for PTU-HPM and from 244% to 466% for PTU-HPM-Zn. This behavior is characterized by a decrease in tensile strength accompanied by an increase in ductility, indicating a reduction in network rigidity after hot pressing. FT-IR analysis confirmed the retention of pyrimidine and urethane structures after reprocessing, suggesting that no obvious chemical degradation occurred during thermal processing (Figure S3, Supporting Information). The mechanical behavior of the reprocessed materials became closer to that of the linear polyurethane PTU-HP, indicating that the network rearrangement occurred during hot pressing, resulting in improved chain mobility and ductility [44]. This result fully confirms the reversibility of the system based on multiple dynamic bonds and verifies its excellent thermal remoldability.

**Figure 8:**
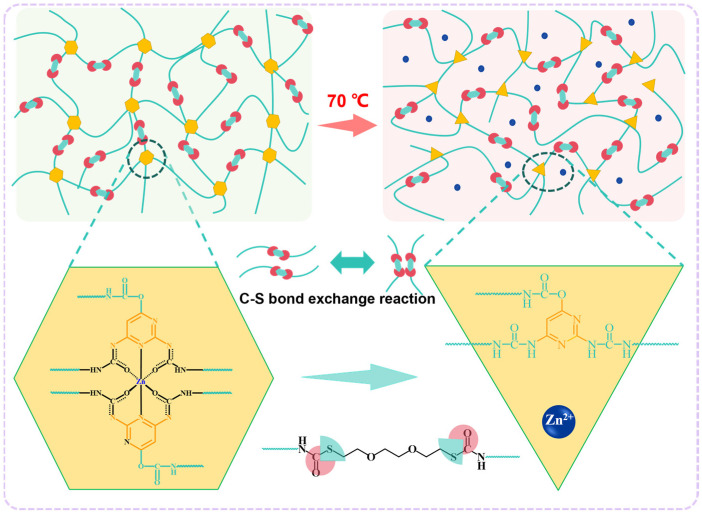
The dynamic kinetic mechanism of PTU-HPM and PTU-HPM-Zn.

Thiourethane bonds with reversible exchange properties were introduced into the system via the thiol–isocyanate click reaction between the chain extender 2,2′-(diethoxy)diethanethiol and polyurethane prepolymer. After fabricating the lightly crosslinked polyurethane PTU-HPM via pyrimidine-based coordination compounds, a denser crosslinked network was further constructed by exploiting the coordination capability of Zn^2+^—specifically, Zn^2+^ is likely to coordinate with N atoms on the pyrimidine ring and may additionally interact with O atoms of adjacent urea groups, as suggested by the simultaneous shifts in the corresponding FTIR bands [52]. This possible multidentate coordination mode could effectively enhance the material’s mechanical strength. The proposed network structure and the possible reversible changes of Zn^2+^ coordination interactions during healing and reprocessing are illustrated in Figure 8. When the material is damaged, its self-healing process mainly relies on the exchange reaction of dynamic thiourethane bonds, but the coordination interaction between Zn^2+^ and pyrimidine still restricts the mobility of molecular chains. Thus, compared with PTU-HPM without effective coordination structure, PTU-HPM-Zn exhibits a slower healing rate at 50 °C. When the healing temperature is increased to 70 °C, the thiourethane bond (bond energy 218 kJ·mol^−1^) shows a significant response to thermal stimulation due to its low bond energy: on the one hand, heating provides sufficient activation energy for polymer segments, effectively enhancing segment mobility and accelerating the dynamic exchange of thiourethane bonds; on the other hand, thermal action can induce partial dissociation of the metal–ligand coordination crosslinked structure, further relieving the spatial constraint of the network on segments, which synergistically improves the efficiency of segment migration and network reorganization. However, after the material is subjected to hot-pressing treatment at 120 °C and 10 MPa, the metal coordination effect is largely disrupted, and the coordination crosslinking sites undergo partial dissociation. This leads to a significant reduction in the crosslinking density of PTU-HPM, resulting in a pronounced decrease in tensile strength and a remarkable increase in elongation at break. This reversible transformation from a highly crosslinked network to a loosely crosslinked state, facilitated by the dynamic nature of both Zn^2+^–pyrimidine coordination bonds and thiourethane exchange reactions, endows the material with excellent reprocessability and potential for cyclic utilization.

## 4. Conclusions

This study adopted a synergistic design strategy of “dynamic thiourethane backbone + Zn^2+^-multidentate pyrimidine coordination”. A polythiourethane system containing multiple dynamic bonds was constructed via thiol–isocyanate click reaction. Combined with the coordination interactions between different functional ligands and Zn^2+^, highly transparent, self-healing, and reprocessable polyurethane elastomers were successfully prepared. The research results show that the multifunctional ligand HPM can form a stable multidentate coordination network with Zn^2+^, which can effectively inhibit microphase separation of the material. Cooperating with dynamic thiourethane bonds, this network achieves an optimized balance of comprehensive material properties: the PTU-HPM-Zn composite exhibits both high tensile strength of 9.04 MPa and excellent light transmittance of 89.0% (at 650 nm), can complete full scratch healing within 4 h at 70 °C, and shows good reprocessability and cyclic utilization potential under hot-pressing conditions. This design strategy effectively addresses the performance trade-off issue between the mechanical strength, transparency, and healing efficiency of traditional self-healing materials, providing new ideas and technical support for the development of intelligent elastomers for high-end optical and flexible electronic applications.

## Data Availability

The original contributions presented in this study are included in the article/Supplementary Material. Further inquiries can be directed to the corresponding author.

## Supplementary Materials

The following supporting information can be downloaded at https://www.mdpi.com/article/10.3390/polym18151910/s1, Figure S1: The GPC spectra of PTU-IZ, PTU-HP, PTU-HPM. Table S1: molecular weight of PTU-IZ, PTU-HP, PTU-HPM. Figure S2: Tensile stress–strain curves of PTU-HPM-Zn before and after healing at 70 °C for 4 h. Figure S3: FT-IR spectra of PTU-HPM (a) and PTU-HPM-Zn (b) before and after hot pressing.
