# Supplementary material for: Constructing High-Transparency, Self-Healing and Reprocessable Poly(thiourethane) Elastomers Based on Zn2+-Multidentate Pyrimidine Coordination
Source: Polymers (Basel). 2026 Aug 4;18(15):1910. doi: 10.3390/polym18151910 (PMC13468476; doi:10.3390/polym18151910)
Supplement: Supplementary file 1 [file polymers-18-01910-s001.zip › polymers-4423917-supplementary.pdf]

Supporting Information:

Constructing High-Transparency, Self-Healing and Reprocessable Poly(thiourethane) Elastomers Based on Zn^{2+}-Multidentate Pyrimidine Coordination

Na Wei [1,†], Hanxu Zhu [1,2,†], Bing Li [1] and Weijun Yang [1,3,4,*]

[1] The Key Laboratory of Synthetic and Biological Colloids, Ministry of Education, School of Chemical and Material Engineering, Jiangnan University, Wuxi 214122, China; 6240608018@stu.jiangnan.edu.cn (N.W.); zhuhanxu@nimte.ac.cn (H.Z.); libing_73@163.com (B.L.)

[2] Key Laboratory of Bio-Based Polymeric Materials Technology and Application of Zhejiang Province, Ningbo Institute of Materials Technology and Engineering, Chinese Academy of Sciences, Ningbo 315201, China

[3] Collaborative Innovation Center of Chemical Science and Engineering, Key Laboratory for Green Chemical Technology of Ministry of Education, School of Chemical Engineering and Technology, Tianjin University, Tianjin 300072, China

[4] Zhejiang Rongsheng Environmental Protection Paper Co., Ltd., No. 588 East Zhennan Road, Pinghu Economic Development Zone, Jiaxing 314213, China

* Correspondence: weijun.yang@jiangnan.edu.cn

† These authors contributed equally to this work.

S1. Experimental section

S1.1 Materials

Zinc chloride ($ZnCl_2$, 98%), 1-(3-aminopropyl)imidazole (IZ, ≥97%), 2,4-diamino-6-hydroxypyrimidine (HPM, ≥98%), isophorone diisocyanate (IPDI, 99%), N,N-dimethylacetamide (DMAc, 99%), and dibutyltin dilaurate (DBTDL, 95%) were purchased from Shanghai Maclin Biochemical Technology Co., Ltd. 3-Hydroxypyridine (HP, 98%), poly(tetramethylene ether) diol (PTMG-1000, M_n= 1000 $g\cdot mol^{-1}$), 2,2'-(diethoxy)diethanethiol (TGLDM, ≥97%), and N,N-diisopropylethylamine (DIPEA, 99%) were obtained from Aladdin Biochemical Technology Co., Ltd. (Shanghai, China). All reagents were used without further purification.

S1.2 Preparation of Polyurethane Films and Zn^{2+}-Coordinated Composites

Synthesis of PTU-IZ, PTU-HP, and PTU-HPM Films: 4.00 g of PTMG was weighed into a three-necked flask, dried under vacuum at 100 °C for 1 h, and then cooled to 70 °C. 1.79 g of IPDI and two drops of DBTDL catalyst were added, followed by dissolving in 3 g of DMAc. The reaction was carried out at 70 °C for 2.5 h under

nitrogen protection to obtain the prepolymer. Subsequently, 0.364 g of TGLDM and two drops of DIPEA catalyst were added, dissolved in 6 g of DMAc, and the chain extension reaction was performed at 65 °C for 8 h to obtain the polythiourethane (PTU) solution. Then, 0.266 g of IZ, 0.380 g of HP, and 0.168 g of HPM were added separately, and the reaction was continued at 60 °C for 12 h. The resulting solution was poured into a mold, volatilized at room temperature for 24 h, and then dried at 50 °C to obtain PTU-IZ film, PTU-HP film, and PTU-HPM film.

Preparation of Zn^{2+}-Coordinated Composites (PTU-IZ-Zn, PTU-HP-Zn, PTU-HPM-Zn): The above three polyurethane films were weighed separately and dissolved in 10 g of DMAc, followed by stirring at 60 °C under nitrogen protection until completely dissolved. According to the molar ratio of Zn^{2+} to ligand of 1:1, the corresponding mass of $ZnCl_2$ was weighed, dissolved in 2 g of DMAc, added to the above solution, and ultrasonically dispersed for 5 min, then stirred at 50 °C for 12 h. The solution was poured into a mold, volatilized at room temperature for 24 h, and dried at 50 °C for 24 h to obtain PTU-IZ-Zn, PTU-HP-Zn, and PTU-HPM-Zn composites, respectively.

S2. Characterizations

S2.1 Fourier Transform Infrared (FT-IR) Characterization

FT-IR measurements were performed on PTU-IZ, PTU-HP, PTU-HPM, PTU-IZ-Zn, PTU-HP-Zn, and PTU-HPM-Zn samples using a Nicolet iS50 FT-IR spectrometer. The scanning range was set from 4000 cm^{-1} to 500 cm^{-1}.

S2.2 Nuclear Magnetic Resonance (^{1} H NMR) Characterization

A total of 5~10 mg of sample was dissolved in deuterated chloroform ($CDCl_3$), and the measurement was carried out on a nuclear magnetic resonance spectrometer with tetramethylsilane (TMS) as the internal standard.

S2.3 Gel Permeation Chromatography (GPC) Characterization

5~10 mg of sample was dissolved in tetrahydrofuran (THF) and allowed to stand for one day to ensure complete dissolution. After filtration, the measurement was performed on a gel permeation chromatography instrument. The sample concentration was 4 mg/mL, and the flow rate was 0.2 mL/min.

S2.4 Ultraviolet–visible spectroscopy (UV-Vis) Measurement

Transparency measurement was conducted on a UV-Vis-NIR spectrophotometer (UV-3600 plus). Dried barium sulfate ($BaSO_4$) was used as the substrate, the measurement mode was set to transmission, and the wavelength range was 200 nm~900

nm. The thickness of both the polyurethane samples and the $BaSO_4$ sheets used for measurement was 0.5 mm.

S2.5 Tensile Test

Samples were cut into specimens with dimensions of 60 mm × 10 mm. The tensile strength and elongation at break were tested on a 5967X universal tensile testing machine, and the maximum tensile strength and maximum elongation at break of the experimental specimens at fracture were recorded. Three sets of samples were tested, and the average value was calculated. The tensile test conditions were set as follows: tensile speed of 50 mm/min.

S2.6 Scratch Healing Test

"X"-shaped scratches were made on the surface of the specimens using a syringe, and the changes in scratch width were tracked and recorded. The changes of scratches on each specimen within 8 h were observed using a VHX-1000C super-depth-of-field 3D microscope. Each specimen was placed at 50 °C, and photographs were taken every 2 h to record the results. Meanwhile, another set of experiments was set up: after making scratches, the changes of scratches on the specimens placed at 70 °C within 8 h were observed, and photographs were taken every 2 h to record the results.

S2.7 Reprocessability Test

Specimens were cut into pieces and subjected to hot-pressing reprocessing in a model vulcanizing machine (press). The test conditions were set as follows: temperature of 120 °C, pressure of 10 MPa, preheating time of 3 min, exhaust count of 10 times, pressing time of 4 min, and cooling time of 3 min.

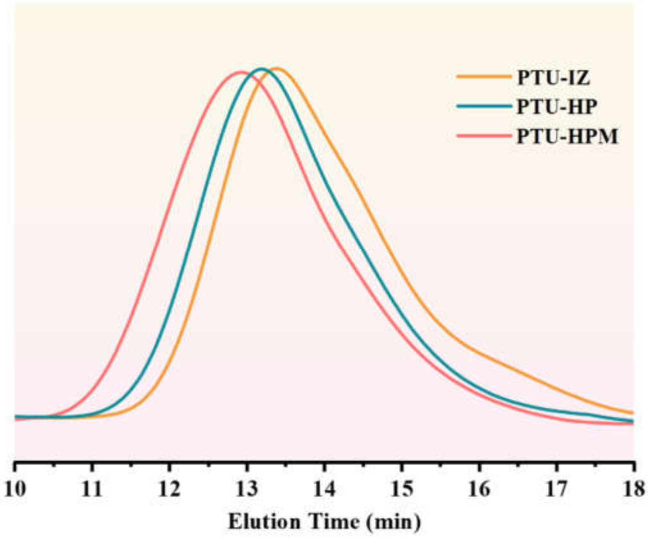

Figure S1: The GPC spectra of PTU-IZ, PTU-HP, PTU-HPM

Table S1: molecular weight of PTU-IZ, PTU-HP, PTU-HPM

samples	Retention time (min)	Mn	Mw	PDI
PTU-IZ	13.40	33500	70400	2.09
PTU-HP	13.17	38960	88550	2.27
PTU-HPM	12.89	47730	90450	1.89

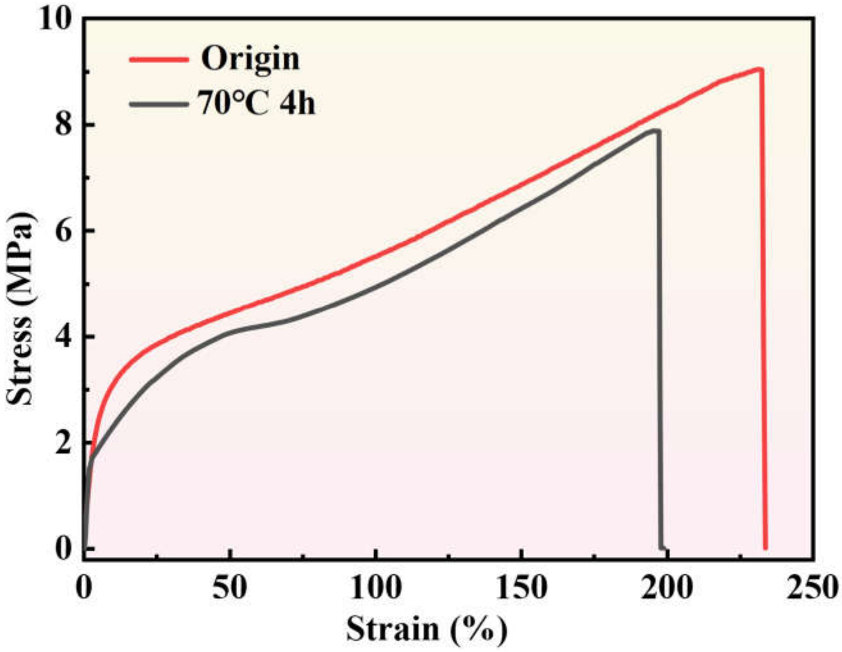

Figure S2: Tensile stress–strain curves of PTU-HPM-Zn before and after healing at 70 °C for 4 h

The self-healing performance of PTU-HPM-Zn was evaluated by tensile recovery tests under healing conditions of 70 °C for 4 h. The healing efficiency (η) was calculated based on the recovery of tensile strength according to the following equation:

$$\eta = \frac{\sigma_h}{\sigma_0} \times 100\%$$

where σ_0 and σ_h represent the tensile strengths of the original and healed samples, respectively.

After the healing treatment, the tensile strength of PTU-HPM-Zn recovered from 9.04 MPa to 7.87 MPa, corresponding to a healing efficiency of 87.1%.

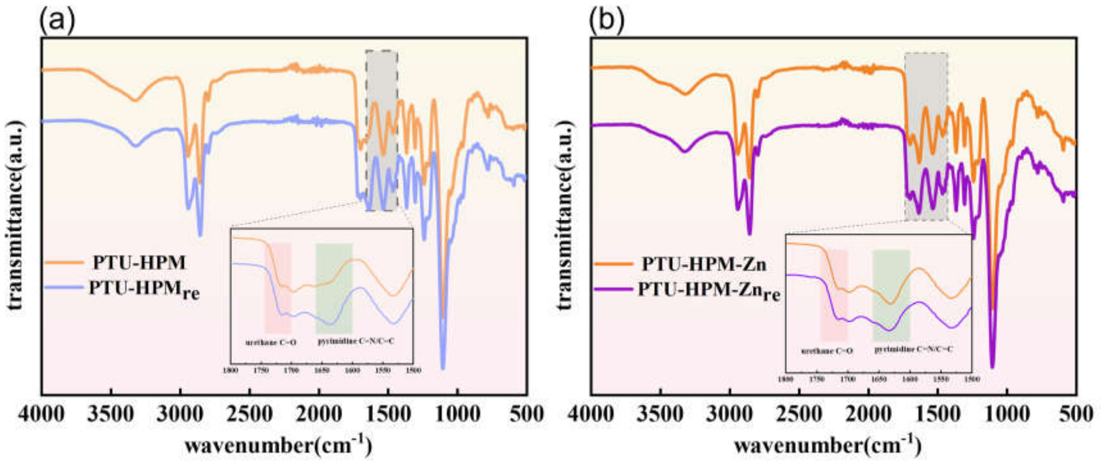

Figure S3. FT-IR spectra of PTU-HPM (a) and PTU-HPM-Zn (b) before and after hot pressing.

To investigate the structural changes during thermal reprocessing, FT-IR spectra of PTU-HPM and PTU-HPM-Zn before and after hot pressing were compared (Figure S3). The characteristic absorption bands associated with pyrimidine structures (C=N/C=C vibrations at 1600–1650 cm^{-1}) and urethane carbonyl groups (1700–1730 cm^{-1}) were retained after reprocessing. Meanwhile, no new absorption bands related to decomposition products were observed.
